# Small molecule and peptide inhibitors of βTrCP and the βTrCP–NRF2 protein–protein interaction

**DOI:** 10.1042/BST20220352

**Published:** 2023-06-09

**Authors:** Uzma Jaffry, Geoff Wells

**Affiliations:** UCL School of Pharmacy, University College London, 29/39 Brunswick Square, London WC1N 1AX, U.K.

**Keywords:** βTrCP, cancer, drug discovery and design, NRF2

## Abstract

The E3 ligase *beta*-transducin repeat-containing protein (βTrCP) is an essential component of the ubiquitin–proteasome system that is responsible for the maintenance of cellular protein levels in human cells. Key target substrates for degradation include inhibitor of nuclear factor kappa B, programmed cell death protein 4 and forkhead box protein O3, alongside the transcription factor nuclear factor erythroid-2-related factor 2 (NRF2) that is responsible for cellular protection against oxidative damage. The tumour suppressive nature of many of its substrates and the overexpression of βTrCP observed in various cancers support a potential therapeutic role for inhibitors in the treatment of cancer. A small molecule substituted pyrazolone, GS143, and the natural product erioflorin have been identified as inhibitors of βTrCP and protect its targets from proteasomal degradation. Modified peptides based on the sequences of native substrates have also been reported with *K*_D_ values in the nanomolar range. This review describes the current status of inhibitors of this E3 ligase. The scope for further inhibitor design and the development of PROTAC and molecular glue-type structures is explored in the context of βTrCP as an example of WD40 domain-containing proteins that are gaining attention as drug targets.

## Introduction

*beta*-Transducin repeat-containing protein (βTrCP) is one of 69 human F-box proteins [1–3] that play roles in regulating the activity of cell cycle proteins and transcription factors. The protein is over expressed in some cancers and has been identified as a regulator of nuclear factor erythroid-2-related factor 2 (NRF2) activity that suggests additional roles in tumour drug resistance, inflammation and redox control. In this review the structure, function and rationale for targeting βTrCP are discussed along with the strategies that have been used to develop small molecule and peptide inhibitors.

## βTrCP

**Figure 1.**
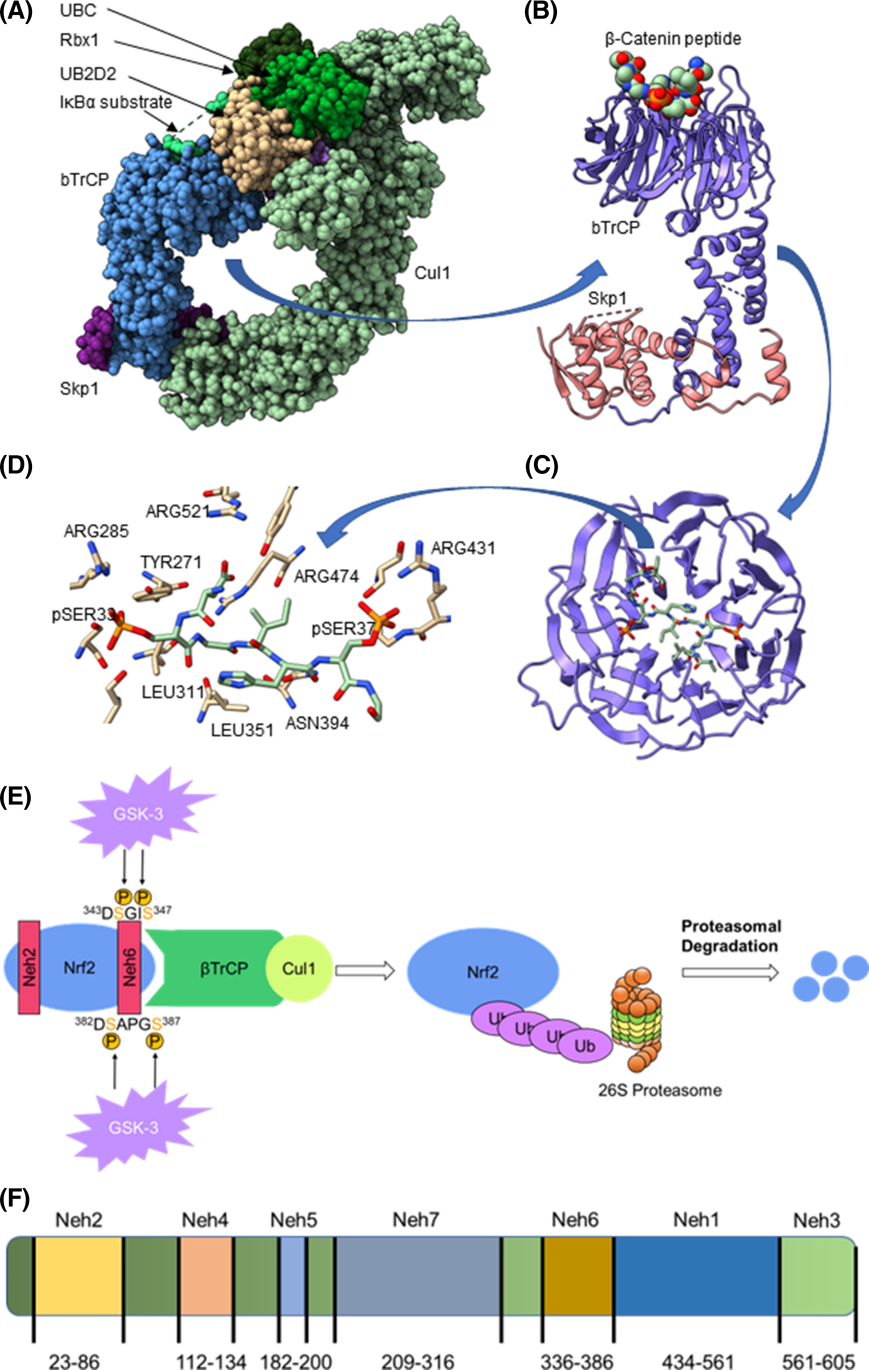
The structure of βTrCP and interactions with binding partners. (**a**) Complex of βTrCP with Skp1, Cul1, RBX1 and an IκBα substrate; (**b**) Complex formed between Skp1 (pink), βTrCP (magenta) and a peptide from β-Catenin (sphere representation); (**c**) WD40 domain of βTrCP in complex with a β-Catenin substrate; (**d**) WD40 domain arginine residues 285, 410, 431 and 510 of βTrCP forming interactions with the β-Catenin phosphopeptide (green backbone); (**e**) Schematic representation of the pathway to substrate degradation mediated by βTrCP using NRF2 as an example; (**f**) Domain structure of Nrf2 with residue boundaries for Neh1-7. The Neh2 domain contains the Keap1 DLG and ETGE degron sequences and the Neh6 domain contains the DSGIS and DSAPGS phosphodegrons.

Human βTrCP has two isoforms, βTrCP1 is a 569 amino acid protein [4] and βTrCP2 has 605 residues [5] (both are referred to as βTrCP in this review). The two isoforms have been considered to be functionally redundant, although a recent study has suggested that there may be subtle differences in activity between the two forms [6]. βTrCP is part of the S-phase kinase-associated protein-1 (Skp1), Cullin-1 (Cul1), F-box protein (SCF) complex SCF^βTrCP^ [7]. The SCF^βTrCP^ complex forms part of a cullin–RING E3 ligase (CRL1), in which Skp1 functions as an adaptor protein, Cul1 acts as a scaffold, βTrCP is responsible for substrate recognition and the RING finger protein RING-box protein 1 (RBX1) participates in ubiquitination as part of the ubiquitin–proteosome system (UPS). The structure of a complex of the proteins in combination with neural precursor cell expressed developmentally down-regulated protein 8 (NEDD8) and an inhibitor of nuclear factor κB-alpha (IκBα) substrate has been solved using cryo-electron microscopy (Figure 1a) [8]. In this complex, the 40-residue F-box region of βTrCP interacts with Skp1 and the WD40 domain interacts with the substrate (Figure 1b). This interaction can also be seen in the structure of βTrCP with Skp1 and a β-Catenin substrate determined by X-ray crystallography (Figure 1b) [9]. The WD40 domain is composed of multiple WD40 repeat (WDR) motifs (runs of ∼40 amino acids terminating with a Tryp-Asp) that consist of a series of β-sheets and are organised into a 7-bladed β-propeller structure (Figure 1c) that has a shallow substrate binding cavity on its upper surface (Figure 1b). This domain recognises consensus degron motifs typically composed of a DpSGXX(X)pS sequence within substrates (Table 1) although a broader group of sequences with either DSG, DDG, EEG, SSG or TSG preceding the variable residues and S, T, E or D following them covers most binding partners [9–11]. The serine residues within the typical DPSGXXS degron motifs usually require phosphorylation to bind effectively to βTrCP and removal of either serine residue results in a large decrease in affinity (Table 2). The phosphorylation of substrate proteins is controlled by specific kinases, including glycogen synthase kinase-3 (GSK-3), that ultimately regulate protein degradation via βTrCP. The phosphopeptide degrons within the substrates form electrostatic interactions with arginine residues Arg^285^, Arg^431^, Arg^474^ and Arg^521^ that surround the βTrCP-binding pocket (Figure 1d) [12–14]. Residues including Leu^351^ and Asn^394^ interact with the central XX(X) motif of the substrates.

**Table 1.** Previously reported bTrCP substrates

Gene code	Gene	Degron sequence (sequence range)
ATF4	Cyclic AMP-dependent transcription factor ATF-4	^215^SDNDSGICMS^224^
BCL2L11	Bcl-2-like protein 11	^90^SRSSSGYFSF^99^
BORA	Protein aurora borealis	^493^IQMDSGYNTQ^502^
CDC25A	M-phase inducer phosphatase 1	^78^ESTDSGFCLD^87^
CDC25B	M-phase inducer phosphatase 2	^265^TEEDDGFVDI^274^
CLSPN	Claspin	^26^SPSDSGQGSY^35^
CPEB1-a	Cytoplasmic polyadenylation element-binding protein 1-A	^187^DSDTSGFSSG^196^
CTNNB1	Β-Catenin-1	^29^SYLDSGIHSG^38^
DEPTOR	DEP domain-containing mTOR-interacting protein	^283^SCGSSGYFSS^292^
DLG1	Disks large homolog 1	^594^KTKDSGLPSQ^603^
eEF2K	Eukaryotic elongation factor 2 kinase	^437^NSGDSGYPSE^446^
ELAVL1	ELAV-like protein 1	^293^TNYEEAAMAI^302^
EPOR	Erythropoietin receptor	^458^VVSDSGISTD^467^
FANCM	Fanconi anaemia group M protein	^940^NVLDSGYNSF^949^
FBXO5	F-box only protein 5	^141^LYEDSGYSSF^150^
FGD1	RhoGEF and PH domain-containing protein 1	^279^PNRDSGIDSI^288^
FZR1	Fizzy and cell division cycle 20-related 1	^137^SSPDDGNDVS^146^
GHR	Growth hormone receptor	^380^KDGDSGRTSC^389^
HNRPD	Heterogeneous nuclear ribonucleoprotein D0	^79^HSNSSPRHSE^88^
IFNAR1	Interferon alpha/beta receptor 1	^531^TSQDSGNYSN^540^
MCL1	Induced myeloid leukemia cell differentiation protein Mcl-1	^154^TSTDGSLPST^163^
MYC	Myc proto-oncogene protein	^275^KRSESGSPSA^284^
NFE2L2	Nuclear factor erythroid 2-related factor 2	^340^NDSDSGISLN^349^
		^381^LDSAPGSVKQ^390^
NFKB1	Nuclear factor NF-kappa-B p105 subunit	^92^3SVCDSGVETS^932^
NFKB2	Nuclear factor NF-kappa-B p100 subunit	^862^VKEDSAYGSQ^871^
NFKBIA	NF-kappa-B inhibitor α	^28^DRHDSGLDSM^37^
NFKBIB	NF-kappa-B inhibitor β	^15^EWCDSGLGSL^2^4
NFKBIE	NF-kappa-B inhibitor ε	^153^SQYDSGIESL^162^
PDCD4	Programmed cell death protein 4	^67^SSRDSGRGDS^76^
PER1	Period circadian protein homolog 1	^118^NPSTSGCSSE^127^
PER2	Period circadian protein homolog 2	^91^NPSTSGCSSD^100^
PLK4	Serine/threonine-protein kinase PLK4	^281^DSIDSGHATI^290^
PRLR	Prolactin receptor	^345^PDTDSGRGSC^354^
REST	RE1-silencing transcription factor	^1005^IDEDEGIHSH^1014^
SIPA1L1	Signal-induced proliferation-associated 1-like protein 1	^1301^TSADSGIDTA^1310^
SNAI1	Zinc finger protein SNAI1 (SNAIL)	^92^SDEDSGKGSQ^101^
SP1	Transcription factor Sp1	^724^LPLDSGAGSE^733^
TP53	Cellular tumor antigen p53	^358^EPGGSRAHSS^367^
TWST1	Twist-related protein 1	^21^PADDSLSNSE^30^
UHRF1	E3 ubiquitin–protein ligase UHRF1	^91^SDTDSGCCLG^100^
USP37	Ubiquitin carboxyl-terminal hydrolase 37	^854^SDEDSGNEDV^863^
VPU	Protein VPU	^48^RAEDSGNESE^57^
WEE1	Wee1-like protein kinase	^49^TGEDSAFQEP^58^
		^113^SWEEEGFGSS^122^
WWTR1	WW domain-containing transcription regulator protein 1	^310^QSTDSGLGLG^319^
YAP1	Transcriptional co-activator YAP1	^396^DESTDSGLSMS^405^

*Notes*: Table adapted from Low *et al.* [10] and Chowdhry et al. [15].

**Table 2.** Binding affinity of selected peptide binding affinity to the βTrCP protein

Entry	Origin of peptide sequence	Sequence	Binding affinity (µM)
Peptides evaluated using STD NMR^1^			
1	ATF4	^208^ IK…DN **D*pS*GICM*pS*** PE…LG^230^	*K*_d_ = 500
2	IκBα	^ 21^ KK…RH **D*pS*GLD-*pS*** MK…EQ^44^	*K*_d_ = 400
3	VPU	^ 41^ LI…AE **D*pS*GNE-*pS*** EG…SA^62^	*K*_d_ = 200
4	β-Catenin	^ 17^ DR…YL **D*pS*GIH-*pS*** GA…SG^48^	*K*_d_ = 1000
Peptides evaluated using fluorescence polarisation^2^			
	Native sequences		
5	β-Catenin	^ 17^ DR…YL **D*pS*GIH-*pS*** GA…SG^48^	*K*_d_ = 0.005
6		^ 17^ DR…YL **D-SGIH-*pS*** GA…SG^48^	*K*_d _> 10
7		^ 17^ DR…YL **D*pS*GIH--S** GA…SG^48^	*K*_d_ = 0.689
8		^ 17^ DR…YL **D-SGIH--S** GA…SG^48^	*K*_d _> 100
	Synthetic sequences^2^		
9	β-Catenin-derived	**D*pS*GIH--S**-NH_2_	IC_50_ > 100
10		G**D*pS*GIH--S**-NH_2_	IC_50_ > 100
11		**D*pS*GIF--E**-NH_2_	IC_50_ = 1.24
12		D-**EGFF****--E**-NH_2_	IC_50_ = 43.6
13		*Suc*-**EGFF--E**-NH_2_	IC_50_ = 3.18
14		*Mps*-***dE*G*F^c^*W-E**-NH_2_	IC_50_ = 0.017
15		*Ts*-***dE*G*F^d^*W-E**-NH_2_	IC_50_ = 0.010

Abbreviations: residues with an –NH_2_ have a *C*-terminal carboxamide; non-standard residues are shown in italics, *pS*, phosphoserine; *dE*, d-glutamic acid; *F^c^* 3-fluorophenylalanine; *F^d^* 3-chlorophenylalanine; *Suc*, succinyl; *Mps*, 4-methoxyphenylsulfonly; *Ts*, *para*-toluenesulfonyl;

1 *K*_d_ values determined using a maltose-binding protein-βTrCP^253−547^ WD40 domain construct;

2 *K*_d_ and IC_50_ values determined using a full length βTrCP-Skp1 complex. The consensus binding sequence for βTrCP is shown in bold for each peptide, dashes are used to align the sequences within the table.

## The role of βTrCP in cancer

More than 50 βTrCP substrates have been identified that contain the DSGXX(X)S sequence in which the serines can phosphorylated to form a phosphodegron, and a further group of candidates have been identified using proteomics methods [10]. Amongst the well-studied substrates are proteins that play roles in the cell cycle, signal transduction, apoptosis, cell migration and tumourigenesis. The role of βTrCP in tumourigenesis may be significant: several of its substrates are tumour suppressors and are inactivated by βTrCP-mediated ubiquitination.

The tumour suppressive substrates include inhibitor of nuclear factor kappa B (IκB), programmed cell death protein 4 (PDCD4) and forkhead box protein O3 (FOXO3). A reduction in IκB concentrations results in activation of the transcription factor NF-κB that has proinflammatory activity and is associated with tumour development [16]. PDCD4 binds to and inhibits the eukaryotic initiation factor-4A helicase (eIF4A) and limits cell transformation through the suppression of cell growth, promotion of apoptosis and blocking translation. A reduction in PDCD4 expression is observed in advanced breast and prostate carcinomas hence it is considered to be a tumour suppressor [12,13]. The down-regulation of transcription factor FOXO3 has been shown in animal models to promote tumourigensis and tumour proliferation [17]. Knocking out βTrCP in breast cancer cell lines reduced cell growth and enhanced the effects of cytotoxic drugs such as doxorubicin and paclitaxel [18]. Additionally, overexpression of βTrCP has been observed in various tumours including those of the colon, pancreas, breast, liver and melanomas [14,19–22]. It has been proposed that this increased activity in cancer cells could result in promoted tumour cell growth and an accelerated cell cycle leading to genetic instability [23]. Overall, the tumour suppressive activity of its substrates and the overexpression of βTrCP observed using *in vitro* and *in vivo* models, and in human cancers support a role in tumorigenic activity, so it may be a potential target for cancer therapeutics [24].

Conversely, there are also protein substrates which are considered characteristically oncogenic, implying that βTrCP also has tumour suppressive roles. These substrates include RE1-silencing transcription factor (REST), mouse double minute-2 (MDM2, and the human homologue HDM2) and β-Catenin. REST is a transcriptional repressor and when silenced, there is an increased transformation of cancerous breast epithelial cells. Additionally, when the capacity of REST to inhibit the phosphoinositide-3-kinase (PI3K) pathway is inhibited in cancer cells due to its degradation through βTrCP activity, this is proposed to result in increased cell transformations [13,24]. The oncogenic substrate β-Catenin is stabilised in various cancers leading to the transcription of several oncogenes such as cyclin-D1 and c-*myc* [23]. MDM2 is an oncogene which negatively regulates the p53 signalling pathway that controls the transcription of genes involved in cell cycle arrest, the promotion of apoptosis and genotoxic stress [24]. Cell cycle regulators Wee1, CDC25A/B and Emi-1 are other known βTrCP substrates [12] which play an oncogenic role, specifically the overexpression and up-regulation of CDC25A/B and Emi-1 has been accounted for in human tumour cell lines and cancers such as ovarian, colorectal and breast cancers [25,26]. The oncogenic activity of these substrates imply βTrCP is tumour suppressive in nature as their degradation via the E3 ligase may prevent tumourigenesis.

Thus, the analysis of βTrCP substrates and its overexpression in some tumours supports a role in tumour development, at least in some contexts. The current balance of opinion in the literature leans toward this view, although the situation is complex and the roles of βTrCP1 and βTrCP2 may differ [12,13,24].

## The βTrCP–NRF2 axis

One of the βTrCP substrates that was identified in recent years is NRF2, a member the cap ‘n’ collar (CNC) subfamily of basic region leucine zipper (bZIP) transcription factors. NRF2 is involved in a range of cytoprotective and anti-inflammatory effects in cells, its biology and potential as a therapeutic target have been extensively reviewed [27–33] and are summarised briefly below.

NRF2 plays a significant role in opposing oxidative stress generated by reactive oxygen and nitrogen species derived from external factors and internal metabolism. Whilst oxidant production usually occurs in a controlled manner, oxidative stress is the result of uncontrolled production leading to cellular damage and in turn, toxicity and disease [34,35]. Under oxidative stress conditions, NRF2 translocates to the nucleus and heterodimerizes with small MAF (sMAF) proteins. These heterodimers subsequently bind to a consensus antioxidant response element (ARE) sequence in the promoter regions of various genes, leading to the activation of a broad pool of target genes and the expression of antioxidant and detoxification enzymes, consequently reducing oxidative stress [35,36]. Because NRF2 activity is associated with reductions in redox stress and anti-inflammatory effects, it has been implicated as a potential therapeutic target in a range of vascular, metabolic and neurodegenerative diseases, and the prevention of certain cancers. Though NRF2 up-regulation is widely considered to be beneficial in normal cells and acts as a blocking agent in the process of carcinogenesis, continuous activation of NRF2 within cancer cells, often caused by mutations in NRF2 or its major regulator Kelch-like ECH-associated protein 1 (KEAP1), leads to tumorigenesis, proliferation and drug resistance. Hence the increased expression of NRF2 in normal cell populations may be desirable, and selective inhibition of its activity in cancerous cells may augment the activity of cytotoxic chemotherapies [36].

Under basal conditions, NRF2 concentrations are kept low through the interaction of ETGE and DLG motifs within its Neh2 domain with a KEAP1 dimer (Figure 1e,f). KEAP1 also functions as an E3 ubiquitin ligase adaptor protein, negatively regulating NRF2 via ubiquitination and consequent proteasomal degradation by the 26S proteasome. However, upon exposure to stimuli, KEAP1 is inactivated through oxidation at reactive cysteine residues allowing for the stabilisation and activation of NRF2 [37–41]. The KEAP1–NRF2 degradation pathway has been extensively investigated as a therapeutic target through the development of electrophiles that interact with KEAP1 cysteine residues and protein–protein interaction inhibitors that occupy the NRF2 binding site of KEAP1 [27,28,30,31,42].

Although KEAP1 is the major regulator, the role of βTrCP in tuning the protein concentrations of NRF2 as an alternative regulatory mechanism has received more attention recently. The interaction with βTrCP is mediated by the conserved peptide motifs DSGIS and DSAPGS within the Neh6 domain of NRF2 (Figure 1e,f), with the former site being more important [15]. Upon phosphorylation of the DSGIS motif by glycogen synthase kinase-3*B* (GSK-3*B*), βTrCP is recruited as part of the SCF ubiquitin ligase complex, ultimately leading to proteasomal degradation of NRF2 (Figure 1e) [15,43]. Unlike the KEAP1–NRF2 pathway, this degradation route has received limited investigation as a target for NRF2 up-regulation and for the effects on its other substrates.

In the following sections the strategies that have been applied to targeting WD40 domain proteins and βTrCP will be reviewed with a focus on peptide and small molecule-based approaches.

## Therapeutic intervention in βTrCP pathways

### Inhibition of WD40 protein domains

WDR domains are prevalent in protein structures, studies suggest that there are between 610 [44] and 921 [45] such proteins in the human genome. They contain between 5 and 8 blades each composed of repeating sequences of 40–60 residues that terminate with a tryptophan–aspartic acid dipeptide [46]. In typical structures containing seven blades, the domain is frequently organised into a β-propeller-type structure with a central cavity that runs through the structure that resembles the hole in a doughnut. The C-terminal domain of βTrCP is typical of this type of structure (Figure 1b,c). The cleft formed by this channel often forms the binding site for the partners that interact with the WDR protein. Some of these sites in WDR proteins have grooves or cavities that are suitable for small molecule interactions, so they present opportunities for therapeutic intervention, but given the abundance of WDR proteins, relatively few have been the focus of drug discovery efforts [46,47].

**Figure 2.**
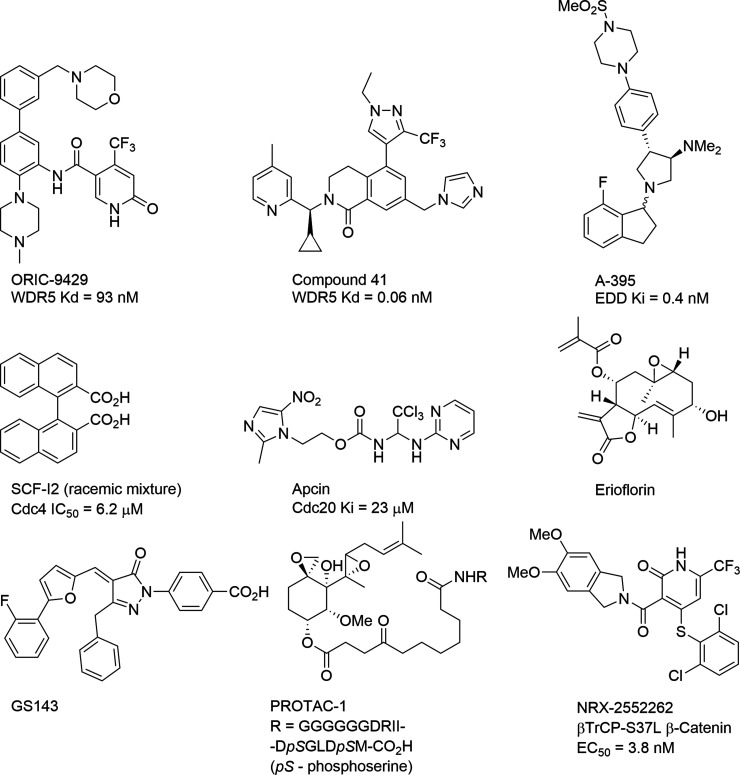
Inhibitors and binders of WD40 domain-containing proteins.

Thus far, four proteins containing WD40 domains have been the focus of ligand discovery efforts: EED [48], Cdc20 [49], WDR5 [47,50] and yeast Cdc4 [46,51] (Figure 2). The WDR5 and EED inhibitors occupy the central substrate binding pocket of each protein. The inhibitors OICR-9429 and Compound 41 bind to WDR5 with affinities of 93 nM and 0.06 nM, respectively [50,52], whilst A-395 binds to EED with a Ki of 0.4 nM [48]. SCF-I2 and Apcin bind to allosteric sites on the outer surface of the Cdc4 and Cdc20 WDRs respectively, although the latter compound is not drug-like and unlikely to be developed further [49,51]. The identification of these ligands suggests that there is potential to target other members of the WDR protein class in humans and organisms that cause infectious diseases for therapeutic applications.

Targeting βTrCP with small molecules has been less successful despite interest in its chemical biology, and its role as a potential cancer target and a ubiquitination facilitator for PROTAC development. In the following sections, the progress to date with approaches to βTrCP inhibition will be discussed.

### Peptide βTrCP inhibitors

Several βTrCP peptide ligands have been synthesised or expressed that are based on known binding motifs and characterised using X-ray crystallography and NMR studies [9,53]. The complexes formed with several peptides were quantified by STD NMR using a maltose-binding protein-tagged βTrCP^253−547^ WD40 domain conjugate. The data indicated that structures with a core DpSGXXpS motif (IκBα, VPU and β-Catenin) and ATF4 with an extended DpSGXXXpS structure all bound relatively weakly to βTrCP with affinities in the range 200–1000 µM (Table 2, entries 1–4) [53]. However, the binding of the βTrCP peptides has also been evaluated using fluorescence polarisation assays using a full length βTrCP protein construct, in this context the peptide with two phosphoserines had a *K*_d_ of 4.8 nM and removal of one or both of the phosphates reduced the binding affinity considerably (Table 2, entries 5–8) [54], suggesting a significant inter-assay variation in binding affinity.

There have been relatively few evaluations of the binding of synthetic peptides to βTrCP. However, a patent filed in 2013 described structure-activity relationships for a large series of short peptides that mimicked the consensus binding motif for βTrCP substrates [55]. The inventors disclosed that truncation of the consensus binding sequence and replacement of both phosphoserines with the isosteric glutamate residues resulted in peptides with micromolar affinities (Table 2, entries 9–12). Substitution of the central isoleucine and histidine with modified phenylalanine and tryptophan residues respectively and the introduction of an *N*-terminal sulphonamide yielded peptides with nanomolar affinities for βTrCP (Table 2, entries 12–15) and activity in cell-based assays. The best sequences included d-amino acids, unnatural amino acids and modifications to the *N*- and *C*-termini that have the potential to improve stability in cellular environments.

### Small molecule βTrCP inhibitors

Similarly, there have also been relatively few studies aimed at discovering small molecule inhibitors of βTrCP. A patent published in 2006 by Genecare Research Institute described a high-throughput screening campaign using 50 000 compounds to identify inhibitors of phosphorylated IκBα ubiquitination [56]. Molecules that caused cellular toxicity were disregarded and a TR-FRET assay was utilised to test the remaining candidates for their ability to inhibit*­* ubiquitination of IκBα *in vitro* using a reconstituted ubiquitin ligase complex containing βTrCP. A supplementary study included synthesis and testing of 569 substituted pyrazalone molecules for their ability to suppress NF-κB activity through inhibition of the E3 ligase [56–58]. Using this approach GS143 (Figure 2) was identified as a lead inhibitor of IκBα ubiquitination with dose-dependent activity and an IC_50_ of 5.2 μM. Interestingly, GS143 activity did not lead to the accumulation of other βTrCP substrates including β-Catenin. Thus, the inhibitory action of the small molecule may be substrate specific [59], suggesting that the mode of action of GS143 warrants further investigation.

The natural product erioflorin (Figure 2), isolated from *Eriophyllum lanatum* has also been identified as an inhibitor of βTrCP using cell-based assays. The compound inhibited the interaction between the tumour suppressive PDCD4 protein and βTrCP, although the mechanism by which this occurs has not been fully elucidated. Erioflorin reduced the degradation of other βTrCP substrates including IκBα and β-Catenin, but not those of other transcription factors such as p21 and HIF-1α. As a consequence of inhibiting the degradation of βTrCP substrates, erioflorin also reduces the activity of downstream transcription factors such as AP-1 and NF-κB and reduced the proliferation of cancer cells [60].

Though advances have been made in the pharmacological targeting of alternate ubiquitin-regulating enzymes in cancer [61], since the discovery of GS143 and erioflorin no other small molecule inhibitors of βTrCP have been identified. Overall, this suggests that there is scope to identify new small molecule inhibitors of βTrCP that may have therapeutic potential as anticancer agents.

## PROTACs and molecular glues

A further area of research where βTrCP-binding molecules are of interest is in the development of PROTACs (proteolysis-targeting chimeric molecules). The use of these conjugate molecules has emerged as a promising technique for the controlled degradation of a protein of interest (POI). PROTACs are linked bifunctional molecules that interact with both an E3 ubiquitin ligase and a POI. The recruitment of the POI to the E3 ligase initiates the catalytic ubiquitination and degradation of the POI [62]. The first PROTAC developed (PROTAC1, Figure 2) utilised βTrCP as the E3 ubiquitin ligase. The PROTAC was composed of an IκBα phosphopeptide (IPP) that binds to βTrCP, linked to the small molecule ovalacin that binds to methionine aminopeptidase-2 (MetAP-2) [63]. Using this approach MetAP-2 was successfully recruited by the PROTAC to SCF^βTrCP^, then degraded following ubiquitination. In this study, the SCF^βTrCP^ was utilised because the ligase complex is constitutively active and therefore the PROTAC targeting approach could be applied to a range of POIs by changing the ligand for the POI. However, as the βTrCP-targeting motif was a phosphopeptide, the compounds had problems associated with drug delivery and stability in the cellular environment. In the absence of a small molecule ligand for βTrCP at the time, subsequent PROTAC development moved to the use of other E3 ligases, including mouse/human double minute-2 (HDM2 or MDM2), von Hippel–Lindau (VHL) tumour suppressor and celebron (CRBN). These approaches have been the subject of many recent reviews of the literature [62,64,65].

A recent study identified small molecules that enhance the interaction between βTrCP and β-Catenin proteins carrying mutations that prevent binding, using the S37A mutation as an example. Compounds such as NRX-2552262 (Figure 2) were able to enhance the interaction between βTrCP and the mutant β-Catenin sequences [54]. Compounds of this type are examples of molecular glues that can enhance or restore interactions between proteins for therapeutic purposes. There may be scope to expand the range of βTrCP-substrate interactions that can be targeted using this approach, this would be particularly interesting for the βTrCP–NRF2 interaction, to reduce NRF2 concentrations in cancer cells [66]. This is a further example of the use of βTrCP as a prototypical E3 ligase for new drug discovery approaches.

## Conclusion

There have been many advances in understanding the structure and functions of WDR domain-containing proteins, and βTrCP in particular, in recent years. βTrCP has a range of substrates that include both tumour suppressors and oncogenes. The current balance of opinion suggests that inhibition of βTrCP [66] activity is likely to have an antitumour effect in some cancers, a notion that is aligned with overexpression of the protein in some cancers. The identification of the role of βTrCP in the regulation of NRF2, a major regulator of redox processes in cells is relatively recent. Efforts to target inhibition of βTrCP with peptides and small molecules have so far been limited to a handful of studies, although promising peptides, and small molecules with activity in ubiquitination assays have been identified. Selective small binders would be of value as chemical probes to assist in understanding the therapeutic potential of targeting βTrCP and would be promising E3 ligase-binding motifs for PROTAC development. Taken together we suggest that further exploration of βTrCP binders is warranted.

## Perspectives

- βTrCP is a WDR-containing F-box protein that is constitutively expressed in human cells. It plays a crucial role in the ubiquitin–proteosome system and acts as a substrate recognition motif for a range of tumour suppressor, oncogenes and the redox-associated transcription factor NRF2.
- The role of βTrCP in cancer is complex due to its diverse range of substrates. However due to its overexpression in cancer cells and its role in the ubiquitination of tumour suppressor proteins, current opinion suggests that it may be an anticancer drug target in some contexts. Its role in regulating NRF2 warrants further investigation to deconvolute the contributions of βTrCP and KEAP1 to its post-translational regulation.
- There are relatively few small molecules and peptides that target WDR-containing proteins in general and βTrCP in particular. Erioflorin and GS143, the two small molecule inhibitors of βTrCP that have been described to date have activity against reconstituted SCF^βTrCP^ but the binding sites and modes of interaction have not been fully described. There is an opportunity to design inhibitors of the βTrCP protein–protein interface that can be investigated as potential antitumour agents and PROTAC E3 ligase-binding motifs.

## Open Access

Open access for this article was enabled by the participation of University College London in an all-inclusive *Read & Publish* agreement with Portland Press and the Biochemical Society under a transformative agreement with JISC.

## Abbreviations

FOXO3: forkhead box protein O3
GSK-3: glycogen synthase kinase-3
HDM2: human double minute-2
IκBα: inhibitor of nuclear factor κB-alpha
KEAP1: Kelch-like ECH-associated protein 1
MDM2: mouse double minute-2
MetAP-2: methionine aminopeptidase-2
NRF2: nuclear factor erythroid 2-related factor 2
PDCD4: programmed cell death protein 4
POI: protein of interest
REST: RE1-silencing transcription factor
UPS: ubiquitin–proteosome system
WDR: WD40 repeat
βTrCP: *beta*-transducin repeat-containing protein
